# Dimensional Stability of Additively Manufactured Dentate Maxillary Diagnostic Casts in Biobased Model Resin

**DOI:** 10.3390/ma17092128

**Published:** 2024-05-01

**Authors:** Münir Demirel, Almira Ada Diken Türksayar, Sandra Petersmann, Sebastian Spintzyk, Mustafa Borga Donmez

**Affiliations:** 1 Department of Prosthodontics, Faculty of Dentistry, Biruni University, Istanbul 34015, Turkey; munirdemirel@biruni.edu.tr (M.D.); aturksayar@biruni.edu.tr (A.A.D.T.); 2 ADMiRE Research Center, Carinthia University of Applied Sciences, 9524 Villach, Austria; s.petersmann@fh-kaernten.at (S.P.); s.spintzyk@fh-kaernten.at (S.S.); 3 Department of Reconstructive Dentistry and Gerodontology, School of Dental Medicine, University of Bern, 3010 Bern, Switzerland; 4 Department of Prosthodontics, Faculty of Dentistry, Istinye University, Istanbul 34010, Turkey

**Keywords:** additive manufacturing, biobased model resin, dental cast, dimensional stability, sustainable manufacturing

## Abstract

This study aimed to evaluate the dimensional stability of maxillary diagnostic casts fabricated from a biobased model resin, which consists of 50% renewable raw materials for sustainable production, a model resin, and stone, over one month. A master maxillary stone cast was digitized with a laboratory scanner to generate a reference file. This master cast was also scanned with an intraoral scanner to additively manufacture casts with a biobased model resin (BAM) and a model resin (AM). Polyvinylsiloxane impressions of the master cast were also made and poured in type III stone (CV) (n = 8). The same laboratory scanner was used to digitize each model one day (T0), 1 week (T1), 2 weeks (T2), 3 weeks (T3), and 4 weeks (T4) after fabrication. Deviations from the reference file were calculated with an analysis software and analyzed with generalized linear model analysis (α = 0.05). The interaction between the material and the time point affected measured deviations (*p* < 0.001). Regardless of the time point, CV had the lowest and AM had the highest deviations (*p* < 0.001). BAM mostly had lower deviations at T0 and mostly had higher deviations at T4 (*p* ≤ 0.011). AM had the highest deviations at T4 and then at T3, whereas it had the lowest deviations at T0 (*p* ≤ 0.002). The measured deviations of CV increased after each time point (*p* < 0.001). BAM casts had deviations within the previously reported clinically acceptable thresholds over one month and had acceptable dimensional stability. Therefore, tested biobased resin may be a viable alternative for the sustainable manufacturing of maxillary diagnostic casts that are to be used clinically.

## 1. Introduction

Computer-aided design and computer-aided manufacturing (CAD-CAM) technologies have facilitated the digitization of intraoral situations by using intraoral scanners (IOSs) or extraoral scanners [1]. Direct digital workflow by using IOSs [2] eliminates technique-sensitive conventional impressions, which are inconvenient for the patients [3,4], and physical models, which require longer duration for fabrication, more space for storage, and are prone to deformation [5,6,7,8]. In addition, a physical cast still can be fabricated by using the digital scan data and either additive or subtractive manufacturing [9,10,11]. However, physical dental casts for either diagnostic or treatment purposes can be fabricated more sustainably with additive manufacturing [3,4,12], given that it facilitates the fabrication of more complex structures with minimum material waste [1]. Three-dimensional (3D) printers with different additive manufacturing technologies are available in the dental market [4,13,14,15,16]; however, those based on liquid crystal display (LCD) technology have started to increase their popularity among dental professionals for the fabrication of dental casts [17] as they are more affordable than 3D printers with other technologies [12].

Even though additive manufacturing can be considered a more ecologically friendly fabrication method compared with subtractive manufacturing [18], the resins used for vat polymerization technologies such as LCD are generally derived from petroleum-based resources [19] and, thus, have increased carbon footprint [20]. Therefore, it is critical to shift towards more sustainable materials for additive manufacturing. An alternative to petroleum-based resins may be the biobased resins that are derived from various sources such as vegetable oil, gelatin, starch, and waste cooking oil [20]. An additive manufacturing resin manufacturer has marketed a biobased model resin (FotoDent biobased model; Dreve Dentamid GmbH, Unna, Germany) that consists of 50% renewable raw materials derived from wheat and corn, which are not involved in food production. Integration of biobased resins into dental applications may facilitate sustainable manufacturing, particularly considering that additive manufacturing is commonly preferred for the fabrication of dental casts [4].

A dental cast should not only be able to replicate the intraoral situation for correct diagnosis but should also have adequate dimensional stability for evaluation over time. However, the accuracy of additively manufactured casts may be impaired due to the uneven accumulation of layers [7]. Previous studies have investigated the fabrication trueness of additively manufactured dental casts [6,7,10,12,13,14,15,21,22,23,24]; however, their dimensional stability has not been investigated broadly [2,9,11]. In addition, the studies focusing on the dimensional stability of additively manufactured casts did not involve biobased resins [2,9,11]. Therefore, the present study aimed to evaluate the dimensional stability of maxillary diagnostic casts fabricated by using an additively manufactured biobased resin by comparing to those of an additively manufactured model resin and conventional stone cast, over one month. The null hypothesis was that the dimensional stability of maxillary diagnostic casts would not be affected by the material type and time point.

## 2. Materials and Methods

### 2.1. Specimen Preparation

**Figure 1:**
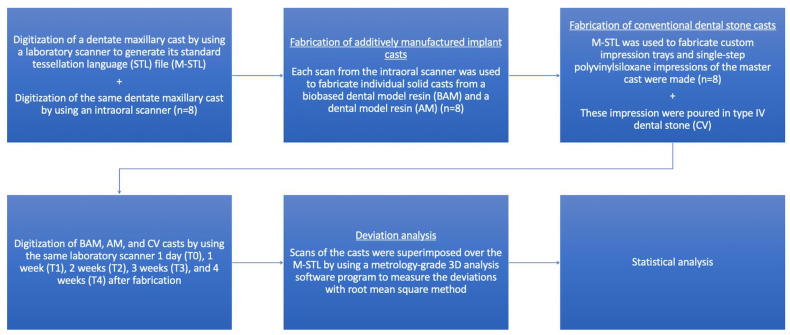
Overview of methodology of this study.

Figure 1 shows the overview of the present study. A priori power analysis based on the results of a previous study on the dimensional stability of additively manufactured casts was performed to determine the number of specimens in each group, and 8 specimens were deemed sufficient (f = 0.84, 1 − β = 95%, α = 0.05) [2]. A dentate maxillary cast was digitized by using a laboratory scanner (inEos X5; Dentsply Sirona, Bensheim, Germany) with high accuracy [25] to generate its standard tessellation language (STL) file (M-STL). Then, a single operator (M.D.) digitized the master model starting from the right second molar by using an IOS (Primescan; Dentsply Sirona, Bensheim, Germany) that was calibrated before each scan for 8 times with the manufacturer’s recommended pattern, and the palate was not scanned. All scans were performed in a room that was daylight-lit and had controlled temperature (23 °C) and humidity (45%). These STL files were imported into a dental design software program (inLab Model SW20.0.1; Dentsply Sirona, Bensheim, Germany) to design digital models with a horseshoe-shaped base and a connecting bar between the right and left second molar teeth [14,15]. The digital model STLs were exported and then imported into a nesting software program (Phrozen 3D; Phrozen Tech Co., Ltd., Hsinchu, Taiwan) and positioned with their base parallel to the build platform. After generating support structures automatically, solid casts were fabricated by using either a biobased dental model resin (FotoDent biobased model; Dreve Dentamid GmbH, Unna, Germany (BAM)) or a dental model resin (FotoDent model 2 beige-opaque; Dreve Dentamid GmbH, Unna, Germany (AM)) (Table 1). An LCD-based 3D printer (Sonic XL 4K; Phrozen Tech Co., Ltd., Hsinchu, Taiwan) [26,27] was used to fabricate all casts with a layer thickness of 100 µm [5,11,12]. After fabrication, all casts were ultrasonically cleaned in 97% isopropyl alcohol for a total of 12 min (2 × 6 min) and left to dry. AM casts were postpolymerized for 8 min, and BAM casts were postpolymerized for 12 min by using the proprietary light-emitting diode polymerization device of the manufacturer (PCU LED N2; Dreve Dentamid GmbH, Unna, Germany) under nitrogen oxide gas atmosphere with 80% light power as recommended [26,27].

The M-STL was imported into a dental design software program (inLab CAD SW22.2.0; Dentsply Sirona, Bensheim, Germany) to design a 3-mm thick custom impression tray with a uniform offset of 2 mm for the impression material. The design file of the tray was imported into a nesting software (inLab CAM SW22.2.0; Dentsply Sirona, Bensheim, Germany) and positioned vertically. After supports were generated automatically, 8 impression trays were fabricated by using an additively manufactured tray resin (Primeprint Tray; Dentsply Sirona, Bensheim, Germany) and a digital light processing 3D printer (Primeprint; Dentsply Sirona, Bensheim, Germany), with a layer thickness of 100 µm. After fabrication, all impression trays were cleaned with 99% isopropanol and postpolymerized under nitrogen oxide gas atmosphere by using the manufacturer’s proprietary unit (Primeprint PPU; Dentsply Sirona, Bensheim, Germany). Eight single-step impressions of the master model were made by the same operator (M.D.) with high- (Elite HD+ Putty Soft; Zhermack, Badia Polesine, Italy) and low-viscosity (Elite HD+ Light Body Fast; Zhermack, Badia Polesine, Italy) polyvinylsiloxane impression material. Each impression was then poured in low expansion (0.04% after 2 h and 0.07% after 48 h) type III stone (Elite Model; Zhermack, Badia Polesine, Italy) that was prepared with a ratio of 30 mL of osmosis water per 100 g of hard dental stone. The stone was first mixed by hand for 60 s and then under vacuum for 30 s. All impression and pouring procedures were performed at room temperature on the same day, and a regular plastic cast base that is in line with American Board of Orthodontics requirements [15] was also used while preparing the casts.

### 2.2. Deviation Analysis

**Figure 2:**
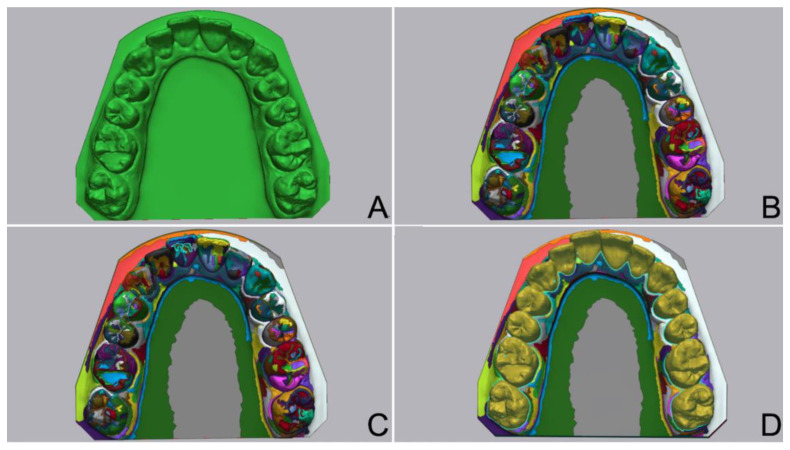
Segmentation process to standardize measured deviations. (**A**) M-STL after importing into analysis software. (**B**) M-STL automatically segmented by analysis software. (**C**) Manual selection of automatically segmented areas for merging. (**D**) M-STL after merging automatically segmented areas to define dental arch.

The same laboratory scanner was used to generate test STLs (T-STL) after storage of one day (T0), 1 week (T1), 2 weeks (T2), 3 weeks (T3), and 4 weeks (T4) in identical lightproof boxes at 23 °C [2]. A metrology-grade 3D analysis software (Geomagic Control X v.2022.1.1; 3D Systems, Morrisville, NC, USA) was used to evaluate the deviations of the casts from the M-STL. M-STL was imported into the software program as the reference file. Then, the “auto segment” feature of the “region tool” of the software was used to automatically segment the entire dental arch on the M-STL. Automatically segmented regions on the dental arch were then merged by using the “merge” feature of the “region tool”, which eliminated any error that might arise from different base designs (Figure 2).

**Figure 3:**
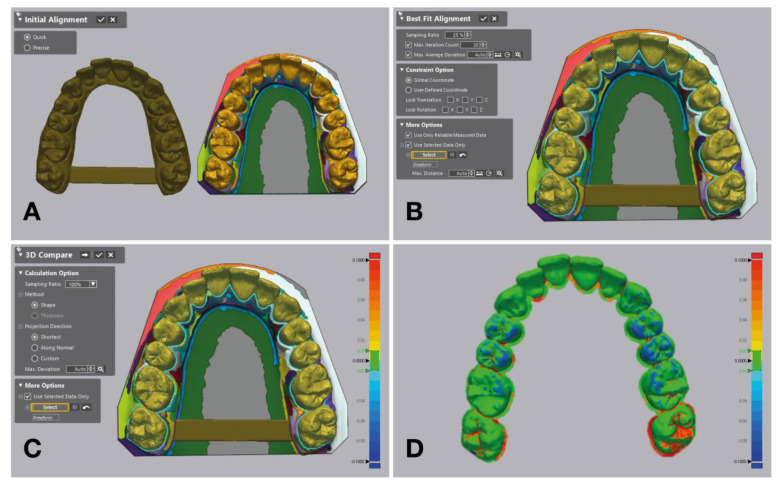
Superimposition of T-STL over segmented M-STL. (**A**) Before initial alignment. (**B**) After best-fit alignment. (**C**) 3D Compare tool of software to generate color maps. (**D**) Generated color map.

**Figure 4:**
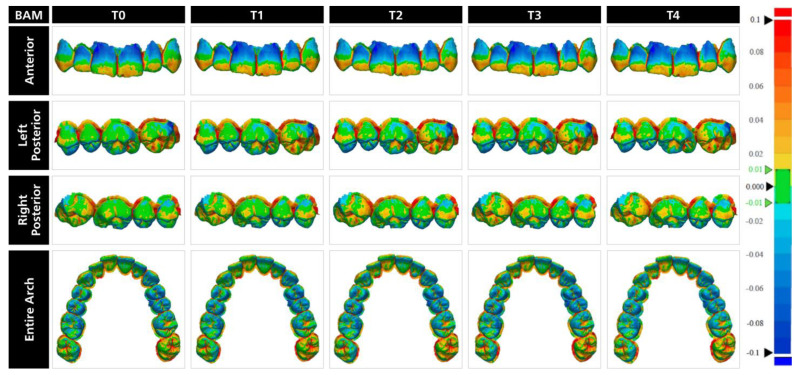
Representative color maps of casts in biobased dental model resin within each time point.

**Figure 5:**
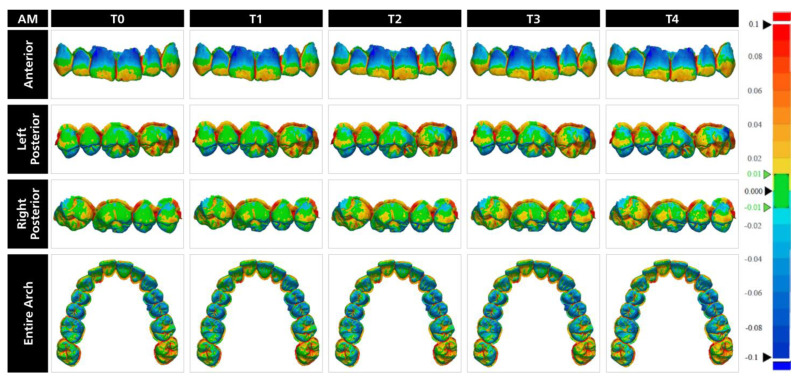
Representative color maps of casts in dental model resin within each time point.

**Figure 6:**
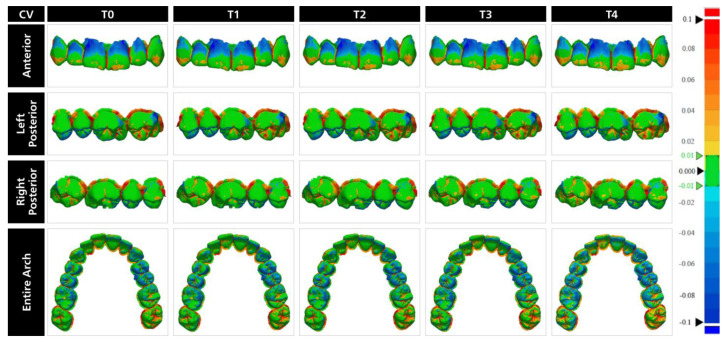
Representative color maps of casts in dental stone within each time point.

T-STLs were superimposed over the M-STL with initial alignment and local best-fit alignment tools of the software program (Figure 3). After superimpositions, the “3D Compare” tool of the software program was used to generate color maps for qualitative evaluation (Figure 4, Figure 5 and Figure 6), and the deviations of each T-STL from the M-STL were automatically calculated by using the root-mean-square method.

### 2.3. Statistical Analysis

The normality of data was analyzed by using the Kolmogorov–Smirnov test. Due to the normal distribution of data, generalized linear model analysis with material type and time point as main factors was performed, and the interaction between these factors was also included. All statistical analyses were performed with a software program (SPSS v25; IBM Corp., Seattle, WA, USA) with a significance level of α = 0.05.

## 3. Results

**Figure 7:**
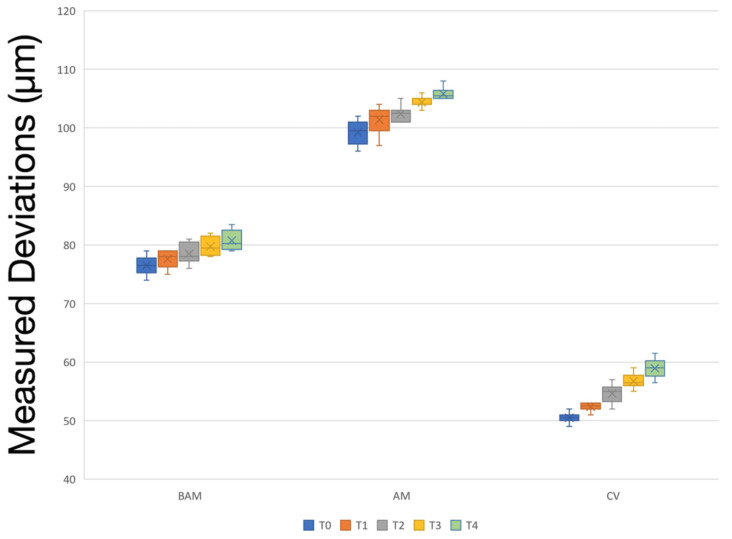
Box plot graph of measured deviations within each resin–time point pair.

Table 2 lists the descriptive statistics of measured deviations within each material–time point pair. Material type, time point, and the interaction between these factors affected the measured deviations (*p* ≤ 0.001). Regardless of the time point, CV had the lowest and AM had the highest deviations (*p* < 0.001). A significant increase in measured deviations was observed among consecutive time points within CV and when pooled data were considered (*p* < 0.001). For BAM, T0 had lower deviations than T2, T3, and T4 (*p* ≤ 0.011). In addition, T1 had lower deviations than T3 and T4, and T2 had lower deviations than T4 (*p* ≤ 0.005). For AM, the lowest deviations were observed at T0, and the highest deviations were observed at T4 followed by T3 (*p* ≤ 0.002) (Figure 7).

## 4. Discussion

The present study investigated the dimensional stability of maxillary diagnostic casts fabricated by using a biobased dental model resin, a dental model resin, and dental stone. Significant differences were observed among the deviation values of tested casts depending on material type and time points. Therefore, the null hypothesis was rejected. Even though CV had the highest and AM had the lowest trueness, it should be emphasized that the maximum mean difference among tested materials was 49 µm (between AM and CV at T1). Considering the size of the casts, differences in these magnitudes may be considered clinically negligible. In addition, the maximum statistically significant mean difference among different time points was just 8.3 µm (between T0 and T4 within CV). Therefore, the authors think that tested materials had similar and acceptable dimensional stability over one month. Nevertheless, the difference between BAM and AM may be associated with the different chemical compositions along with different durations of polymerization. BAM and AM casts were fabricated according to the manufacturer’s printing and postprocessing protocols; however, given that AM casts were polymerized 4 min lesser, these casts might have a lower degree of polymerization and, therefore, higher instability than BAM casts.

The qualitative interpretation of the color maps is essential to identify the possible clinical outcomes of measured deviations and to interpret the differences among tested materials. When the color maps were evaluated from the occlusal aspect, BAM and AM casts mostly had blue color, which represents undercontours, on the occlusal surfaces of the first molars and premolars, and the palatal surface of anterior teeth. An interesting finding when the color maps of BAM and AM casts were analyzed was that the occlusal surface of the left second molar was prominently red and yellow, which represents overcontours. This may be related to the inherent error of the scanning process caused by the IOS and the operator as this tooth was last to be scanned with the IOS. For CV casts, green was the dominant color on the occlusal surfaces with visible blue color at the occlusal surface of premolars, mesioocclusal surface of first molars, and palatal surface of canines. However, unlike BAM and AM casts, the ratio of blue color gradually increased over time and became more evident. This is parallel to the measured deviations over one month as the magnitude of difference between T0 and T4 was higher for CV casts and may be related to the shrinkage of the dental cast. Based on these findings, it can be hypothesized that CV casts may replicate static occlusion more accurately than BAM and AM casts regardless of the time point, while BAM and AM casts would have more stable occlusal contacts over time. However, regardless of the material and time point, undercontoured areas on the occlusal surface might be misleading while analyzing dynamic occlusion.

Regardless of the material and time point, the cervical third of central and lateral incisors were in blue color, with a higher ratio for BAM and AM casts. In addition, yellow, which represents slight overcontours, was more prominent on the incisal third of the anterior teeth in BAM and AM casts. However, for CV casts, green was the evident color with small yellow-colored areas. Therefore, it can be hypothesized that esthetic evaluation of the anterior teeth while using BAM and AM casts could be misleading, and CV casts ensure a more reliable evaluation over one month. When the buccal surfaces of posterior teeth were evaluated, BAM and AM casts had similar color distribution with predominant green color along with yellow and blue at some regions. However, the buccal surfaces of posterior teeth in the color maps of CV casts were more homogenous as colors other than green had a relatively small ratio. The stable color distribution across the vestibular surface of CV casts may indicate that shrinkage varies according to the geometry of the surface, with more complex occlusal and palatal surfaces showing more shrinkage.

Among the limited number of studies on the dimensional stability of additively manufactured casts [2,9,11], only two had also evaluated the dimensional stability over one month with weekly intervals [2,11]. Joda et al. [2] investigated only one dental model resin and showed a gradual increase in measured deviations after each week that was significant after the third and the fourth weeks. The other study on the dimensional stability of additively manufactured casts over the course of one month tested a nanographene-reinforced and a conventional dental model resin against dental stone with a similar methodology and concluded that material type and time point did not affect the stability of the entire dental arch [11]. However, there are some differences between the present and those studies [2,11] such as tested model resin, 3D printer, scanner used for digitization, and the design of the test specimens. In the other study on the dimensional stability of additively manufactured casts, it was reported that the printer type affected measured deviations, whereas storage condition and storage time did not [9]. The deviations of additively manufactured casts were reported to range between 3.3 µm and 579 µm, while the threshold of acceptable deviation of a cast to be used for prosthetic applications was reported to be 200 µm in a recent systematic review [4]. The casts tested in the present study had deviations within these ranges regardless of the time point; therefore, it can be hypothesized that tested model resins are also suitable for prosthetic purposes. However, this hypothesis should also be substantiated with future studies, particularly for biobased dental model resin, as dental casts for prosthetic purposes also involve removable dies, and their dimensional stability could affect their fit into the cast.

Even though the present study was the first on the dimensional stability of additively manufactured casts in biobased dental model resin, the fact that only 1 biobased model resin and 1 dental model resin tested in the present study was a limitation. These resins were deliberately selected as they were both compatible with the tested 3D printer, which standardized the effect of the tested 3D printer on measured deviations. However, another 3D printer with the same or different technology may affect the results. In addition, standardized printing parameters were tested, and different printing orientations [23] and layer thicknesses [8] may affect the results. Given that CV casts were the control group in the present study, they were fabricated with custom-made impression trays and polyvinylsiloxane impression material to have high fabrication trueness. However, in clinical situations, irreversible hydrocolloid impressions with stock impression trays are also made for diagnostic casts; thus, the deviations of CV casts might be higher depending on the impression material used. The methodology of the present study, which involved digitization of tested casts with a laboratory scanner, storage period of one month, and storage in lightproof boxes for standardization, was adopted from a recent study on the dimensional stability of additively manufactured maxillary dentate casts [11]. However, all these factors may affect the measured deviations. BAM and AM casts were manufactured with a horseshoe-shaped solid base with a bar, and different base designs [4] may affect the measured deviations. To simulate an actual clinical situation, the master model was digitized 8 times with a high-accuracy IOS [28]. Even though this methodology also eliminated the bias against polyvinylsiloxane impressions that were repeated 8 times, IOSs’ inherent inaccuracy might affect measured deviations. Patient-related factors could not be simulated in the present study. A metrology-grade software program, referred to by the International Organization for Standardization standard 12836 [29], was used to evaluate the deviations of fabricated casts from the M-STL. However, there are other 3D analysis software programs available, which may affect measured deviations [30]. Finally, the present study evaluated the dimensional stability of dental casts fabricated from a master typodont model that represented mild crowding, and a recent study has shown the effect of anatomical features on the accuracy of dentate casts [16]. Future studies should broaden the findings of the present study with different clinical situations that involve edentulous areas, prepared teeth, dental implants, and shorter spans to elaborate the limitations of additively manufactured casts, particularly those fabricated by using tested biobased dental model resin.

## 5. Conclusions

Regardless of the time point, additively manufactured diagnostic casts in biobased model resin had higher trueness than those in the tested model resin and had lower trueness than those in dental stone, while tested casts had higher deviations after each time point. However, measured deviations were within the previously reported clinically acceptable thresholds. In addition, given that the maximum meaningful mean difference among tested materials was 49 µm and the greatest meaningful mean difference among time points was 8.3 µm, the biobased model resin can be considered as a viable and stable alternative to fabricate diagnostic casts.

## Figures and Tables

**Table 1:** Chemical composition of model resins tested in this study.

Material	Chemical Composition
BAM (Biobased dental model resin; FotoDent biobased model)	Acrylic oligomer: ≥50% Fatty acids, C18-unsatd., dimers, polymers with acrylic acid and 1,3,5-tris(2-hydroxyethyl)-1,3,5-triazine-2,4,6(1H,3H,5H)- trione: ≥1–<10%
AM (Dental model resin; FotoDent model 2 beige-opaque)	Bisphenol A, ethoxylated, dimethacrylate: ≥50% 2-hydroxyethyl methacrylate: ≥1–<6.3% 7,7,9(7,9,9)-trimethyl-4,13-dioxo-3,14-dioxa-5,12-diazahexadecane-1,16-diylbismethacrylate: 2.5–10% Hydroxypropyl methacrylate: ≥2.5–<10% Aliphatic urethane methacrylate: ≥1–<10% Hydroxylpropyl methacrylate: ≥1–<10% Acrylic resin: ≥1–<3.6% Diphenyl(2,4,6-trimethylbenzoyl)phosphine oxide: ≥1–<3% Propylidynetrimethanol, ethoxylated, esters with acrylic acid: ≥0.1–<1%

**Table 2:** Mean ±standard deviation (95% confidence intervals) of deviation values (µm) within each material–time point pair.

	Time Points					
	T0	T1	T2	T3	T4	Total
BAM	76.5 ± 1.6 ^a^^ (75.2–77.8)	77.6 ± 1.5 ^ab^^ (76.4–78.9)	78.5 ± 1.8 ^bc^^ (77.0–90.0)	80.0 ± 1.6 ^cd^^ (78.4–81.1)	80.8 ± 1.7 ^d^^ (79.3–82.2)	78.6 ± 2.2 ^B^ (77.9–79.3)
AM	99.3 ± 2.1 ^a#^ (97.5–101.0)	101.4 ± 2.3 ^b#^ (99.4–103.3)	102.4 ± 1.4 ^b#^ (101.2–103.6)	104.4 ± 0.9 ^c#^ (103.6–105.1)	105.8 ±1.0 ^d#^ (104.9–106.9)	102.6 ± 2.8 ^C^ (101.7–103.5)
CV	50.5 ± 0.9 ^a^* (49.7–51.3)	52.4 ± 0.7 ^b^* (51.8–53.0)	54.6 ± 1.6 ^c^* (53.3–56.0)	56.8 ± 1.3 ^d^* (55.7–57.8)	58.8 ± 1.7 ^e^* (57.4–60.2)	54.6 ± 3.3 ^A^ (53.6–55.7)
Total	75.4 ± 20.4 ^A^ (66.8–84.0)	77.1 ± 20.5 ^B^ (68.5–85.8)	78.5 ± 20.0 ^C^ (70.1–86.9)	80.3 ± 19.9 ^D^ (71.9–88.7)	81.8 ± 19.6 ^E^ (73.6–90.1)	

Different superscript lowercase letters indicate significant differences among time points within each material, while different superscript symbols indicate significant differences among materials within each time point. Different superscript uppercase letters indicate significant differences among the pooled data of materials or time points. Total values are derived from the pooled data of deviations from each material and each time point (*p* < 0.05).

## Data Availability

The data presented in this study are available on request from the corresponding author.
